# Potential impact of cuproptosis-related genes on tumor immunity in esophageal carcinoma

**DOI:** 10.18632/aging.205391

**Published:** 2023-12-30

**Authors:** Pengfei Guo, Zemiao Niu, Dengfeng Zhang, Fangchao Zhao, Jing Li, Tianxing Lu, Xuebo Qin, Shiquan Liu, Zhirong Li, Yishuai Li, Shujun Li

**Affiliations:** 1 Department of Thoracic Surgery, The Second Hospital of Hebei Medical University, Shijiazhuang, China; 2 Graduate school of Hebei Medical University, Shijiazhuang, China; 3 Department of Thoracic Surgery, Hebei Chest Hospital, Shijiazhuang, China; 4 Department of Thoracic Surgery, Affiliated Hospital of Chengde Medical University, Chengde, China; 5 Clinical Laboratory Center, The Second Hospital of Hebei Medical University, Shijiazhuang, China

**Keywords:** esophageal carcinoma, cuproptosis-related genes, cuproptosis, immune contexture, prognostic model

## Abstract

Cuproptosis involves a direct interaction with the tricarboxylic acid (TCA) lipid acylation components. This process intricately intersects with post-transcriptional lipid acylation (LA) and is linked to mitochondrial respiration and LA metabolism. Copper ions form direct bonds with acylated DLAT, promoting DLAT oligomerization, reducing Fe-S cluster proteins, and inducing a protein-triggered toxic stress response that culminates in cell demise. Simultaneously, the importance of immune contexture in cancer progression and treatment has significantly increased. We assessed the expression of cuproptosis-related genes (CRGs) across TCGA and validated our findings using the GEO data. Consensus clustering divided esophageal cancer (ESCA) patients into two clusters based on the expression of 7 CRGs. We evaluated the expression of immune checkpoint inhibitor (ICI) targets and calculated the elevated tumor mutational burden (TMB). Weighted gene co-expression network analysis (WGCNA) identified genes associated with the expression of CRGs and immunity. Cluster 1 exhibited increased immune infiltration, higher expression of ICI targets, higher TMB, and a higher incidence of deficiency in mismatch repair-microsatellite instability-high status. WGCNA analysis identified 14 genes associated with the expression of CRGs and immune scores. ROC analysis revealed specific hub genes with strong predictive capabilities. The expression levels of SLC6A3, MITD1, and PDHA1 varied across different pathological stages; CCS, LIPT2, PDHB, and PDHA1 showed variation in response to radiation therapy; MITD1 and PDHA1 exhibited differences related to the pathological M stages of ESCA. CRGs influence the immune contexture and can potentially transform cold tumors into hot tumors in ESCA patients.

## INTRODUCTION

Esophageal carcinoma (ESCA) is globally recognized as the sixth most prevalent cancer, with a low survival rate [1]. Tumor recurrence and metastasis are identified as the primary causes of mortality among ESCA patients; however, the underlying mechanisms remain unclear [2]. ESCA exhibits significant variations in incidence, mortality, and histopathology across different geographic regions, especially in Southeast Asia/Africa and East Asia, contributing to a significant disease burden [3]. The two main histologic subtypes of ESCA are esophageal adenocarcinoma (EAC) and esophageal squamous cell carcinoma (ESCC), which together constitute over 95% of all ESCA cases [4]. Significant progress has been achieved in the treatment of ESCA in recent decades [5]. Combined approaches, including surgery, chemotherapy, and radiotherapy, have led to improved overall survival rates for patients with this disease [6]. However, a substantial number of ESCA patients are diagnosed at an advanced stage, frequently with delays in initial detection, resulting in a relatively poor prognosis and contributing to over 500,000 annual fatalities [7].

Copper, an essential micronutrient, plays a vital role in fundamental biological processes in all organisms. Being a redox-active metal, copper has the ability to donate and accept electrons, facilitating its transition between the reduced (Cu^+^) and oxidized (Cu^2+^) states [8]. Disruptions in copper homeostasis can lead to cellular toxicity, and changes in intracellular copper levels have been implicated in cancer development and progression [9]. Various copper ion carriers, including disulfide shimron, dithiocarbamate ester, chlorine compounds, and copper chelating agents like Sanchoishi and tetrathiomolybdenate, have been employed in cancer treatment using this mechanism [10–12]. Cuproptosis, a unique pathway, has become a significant player in the development of various cell death mechanisms, including apoptosis, pyroptosis, necroptosis, and ferroptosis [13]. The interaction between copper and the tricarboxylic acid (TCA) cycle influences the lipid acylation process, resulting in protein aggregation during acylation and the depletion of iron-sulfur cluster proteins. This leads to the accumulation of stress-inducing proteins and, ultimately, cell death [14]. Given copper’s dual function as both an essential enzyme cofactor and a potential inducer of cellular toxicity, it shows potential as an innovative therapeutic target. The accumulation of intracellular copper can be precisely targeted to eliminate cancer cells. Reports suggest that the combination of copper with platinum-based antitumor compounds can overcome drug resistance and function as a synergistic radiotherapeutic agent in cancer treatment [15].

Further research has unveiled a strong connection between cuproptosis and esophageal carcinoma. Irregular copper buildup is a common occurrence in different cancer types, which establishes a link between copper levels and cancer advancement [16, 17]. Copper plays a crucial role in promoting tumor growth and angiogenesis, acting as a cofactor for multiple pro-angiogenic molecules, including vascular endothelial growth factor (VEGF) [18]. Moreover, the copper ion plays an active role in the BRAF signaling pathway in cancer, promoting the proliferation and migration of tumor cells [19]. Notably, specific copper chelating agents and inhibitors, like ATOX1 and CCS, have shown the capacity to hinder the proliferation of various cancer cell types [18]. Tetrathiomolybdenate (TM), a copper chelating agent, has demonstrated good tolerability in both animal models and clinical trials, proving its effectiveness in inhibiting angiogenesis and tumor growth [20]. Therefore, the use of copper strips is considered a promising therapeutic approach for treating copper-rich cancers [21]. Nonetheless, the specific genes’ roles in cuproptosis and esophageal cancer have not been investigated. In this study, we pioneer the use of bioinformatics analysis to explore gene differences and assess the impact of genetic variations using gene enrichment analysis.

## RESULTS

### Expression and consensus clustering of CRGs in ESCA

**Figure 1:**
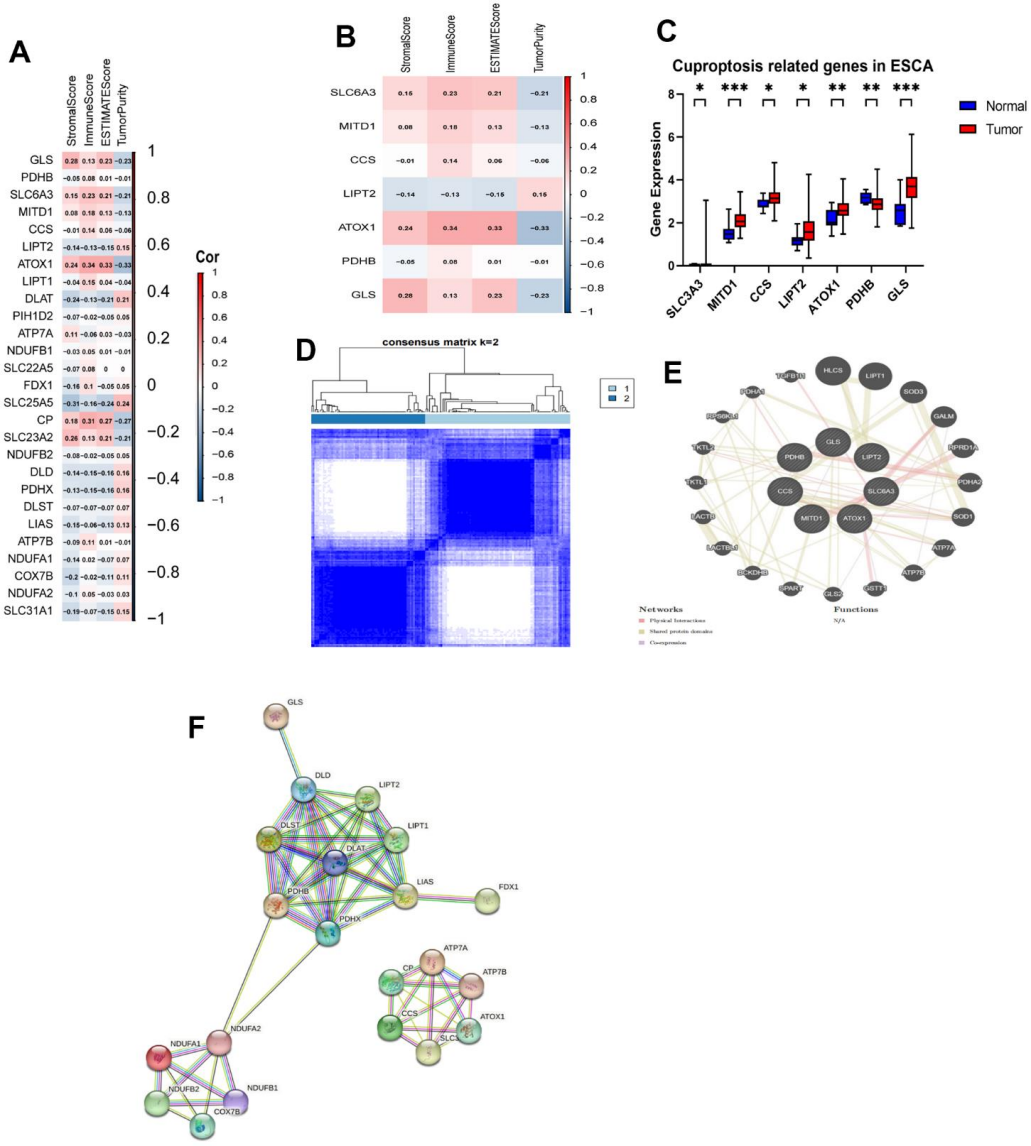
**Identification of CRGs and their association with the immune score and clustering of TCGA-ESCA.** (**A**) Association between 27 CRGs and the ESTIMATE results. (**B**) Association between 7 CRGs and the ESTIMATE results. (**C**) Comparison of expression levels of the 7 CRGs in tumor and normal tissues. (**D**) TCGA-ESCA patients were classified into two clusters based on their expression levels of the 7 CRGs. (**E**) A PPI network of the differentially expressed genes associated with the 7 CRGs. (**F**) A PPI network of the differentially expressed genes associated with the 27 CRGs. *P < 0.05, **P < 0.01, ***P < 0.001.

We performed a comparative analysis of the expression levels of 27 CRGs using a dataset that included 11 normal tissues and 152 tumor tissues from the TCGA database. For evaluating the proportions of stromal and immune cells in each sample, we computed four ESTIMATE indices. We aimed to investigate the role of CRGs in tumor immunity among ESCA patients. We analyzed the correlation between regulator expression and the ESTIMATE results (Figure 1A, 1B). Out of the identified regulators, six genes (SLC6A3, MITD1, CCS, LIPT2, ATOX1, and GLS) showed increased expression in tumor tissues, while PDHB had higher expression in normal tissues. The remaining 20 genes did not display significant differences in expression (Figure 1C). Next, we conducted a consensus clustering analysis on the expression matrix of CRGs using the 152 TCGA-ESCA samples. This analysis led to the classification of samples into two distinct clusters (Figure 1D and Supplementary Figures 1, 2). To explore the interactions among the 27 CRGs, we created a PPI network using online tools available on the STRING and GeneMANIA websites. The network analysis uncovered strong associations among the seven CRGs (Figure 1E, 1F).

### Immunity and pathway enrichment analysis

**Figure 2:**
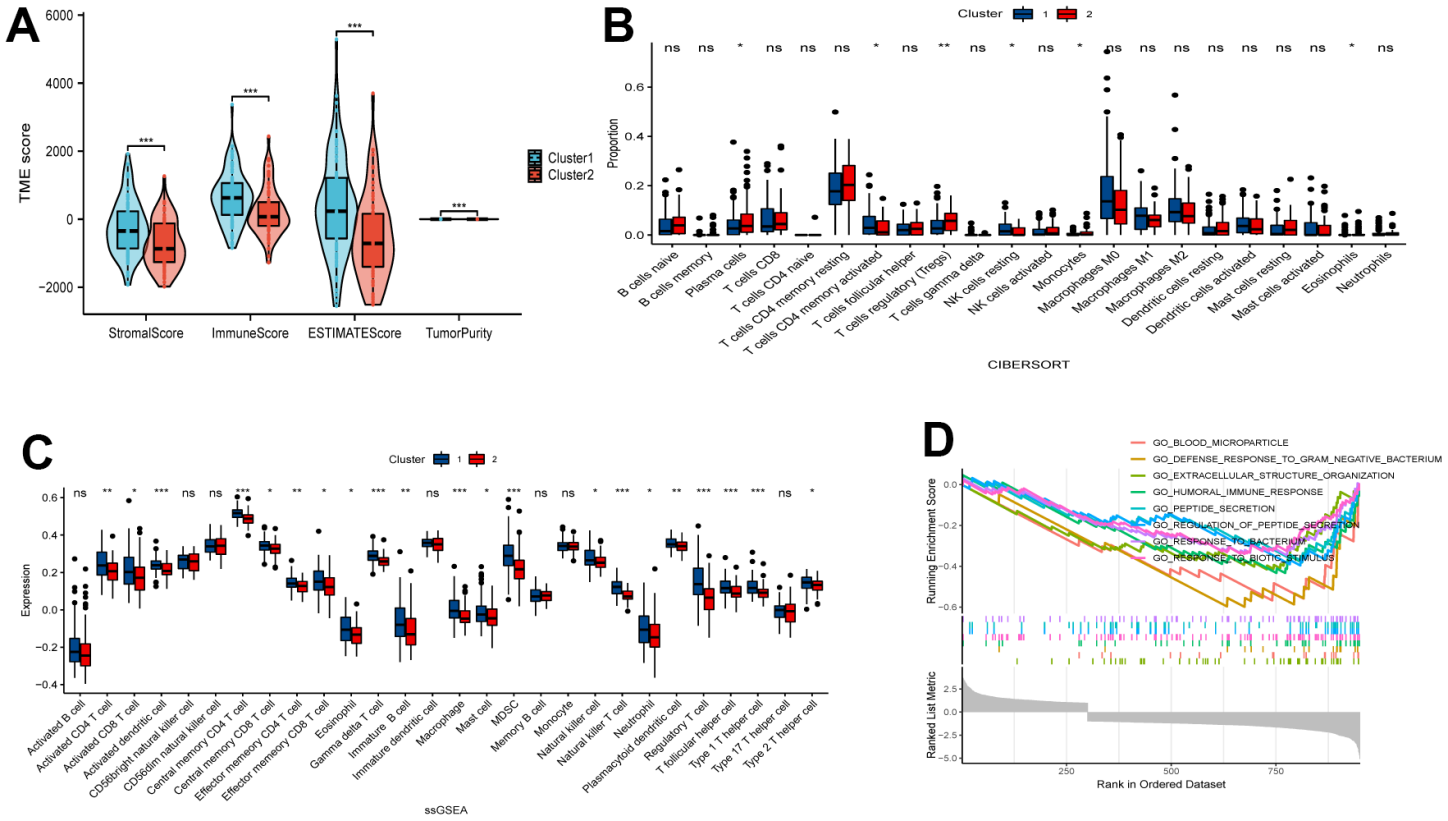
**Comparative analysis of immune characteristics between two clusters.** (**A**) Expression level of stromal score, immune score, ESTIMATE score and tumor purity between the Cluster 1 and Cluster 2. (**B**) Comparison of immune cell proportions and (**C**) expression of immune cells between the two clusters. (**D**) Comparative analysis of functional enrichment. ns, not significant, *P < 0.05, **P < 0.01, ***P < 0.001.

We employed ESTIMATE, CIBERSORT, and ssGSEA analyses to understand the differences in immunological function between the two clusters. Results from the ESTIMATE algorithm showed that Cluster 1 had higher scores in stromal, immune, and estimate parameters and lower tumor purity compared to Cluster 2 (Figure 2A). Additionally, the CIBERSORT analysis revealed a higher proportion of CD4 T cells in Cluster 1 (Figure 2B). Conversely, ssGSEA analysis revealed increased expression of 25 immune cell subtypes, including immature B cells, macrophages, myeloid-derived suppressor cells (MDSCs), plasmacytoid dendritic cells, T follicular helper cells, and type 1 T helper cells, in Cluster 2 (Figure 2C). To explore the functional differences between the two clusters, we conducted GSEA with all the differentially expressed genes from Cluster 1 and Cluster 2. Importantly, we found several significant pathways related to immunity in the enrichment analysis using the MSigDB collection, one of which is the humoral immune response (Figure 2D).

### Evaluation of sensitivity to immunotherapy

**Figure 3:**
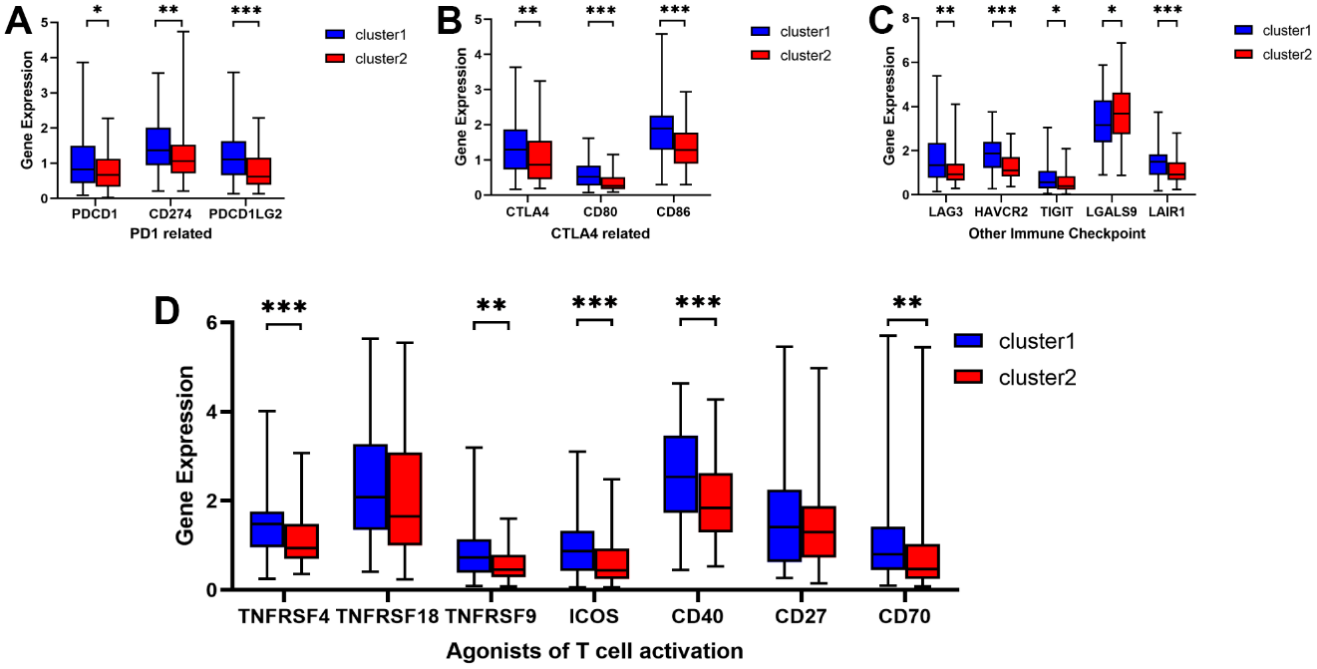
**Comparative analysis of targets of immunomodulatory drugs in clinical trials for metastatic esophageal cancer between two clusters.** The expression levels of immunomodulatory targets related to PD1 (**A**), CTLA4 (**B**), other immune checkpoint molecules (**C**), and agonists of T cell activation (**D**) varied between Cluster 1 and Cluster 2. *P < 0.05, **P < 0.01, ***P < 0.001.

To assess the potential responsiveness of ESCA patients to immunotherapy, we examined the immunomodulatory drug targets identified in clinical trials for metastatic esophageal cancer. Then, we compared the expression levels of these immunomodulatory targets between the two clusters. Remarkably, we observed significantly higher expression of most targets, including PDCD1, CD274, PDCD1LG2, CTLA4, CD80, CD86, LAG3, HAVCR2, TIGHT, LGALS9, LAIR1, TNFRSF4, TNFRSF9, ICOS, CD40, and CD70, in Cluster 1 (Figure 3A–3D). These findings suggest that Cluster 1 may exhibit a more favorable response to immunotherapy than Cluster 2.

### Mutation analysis of CRGs

**Figure 4:**
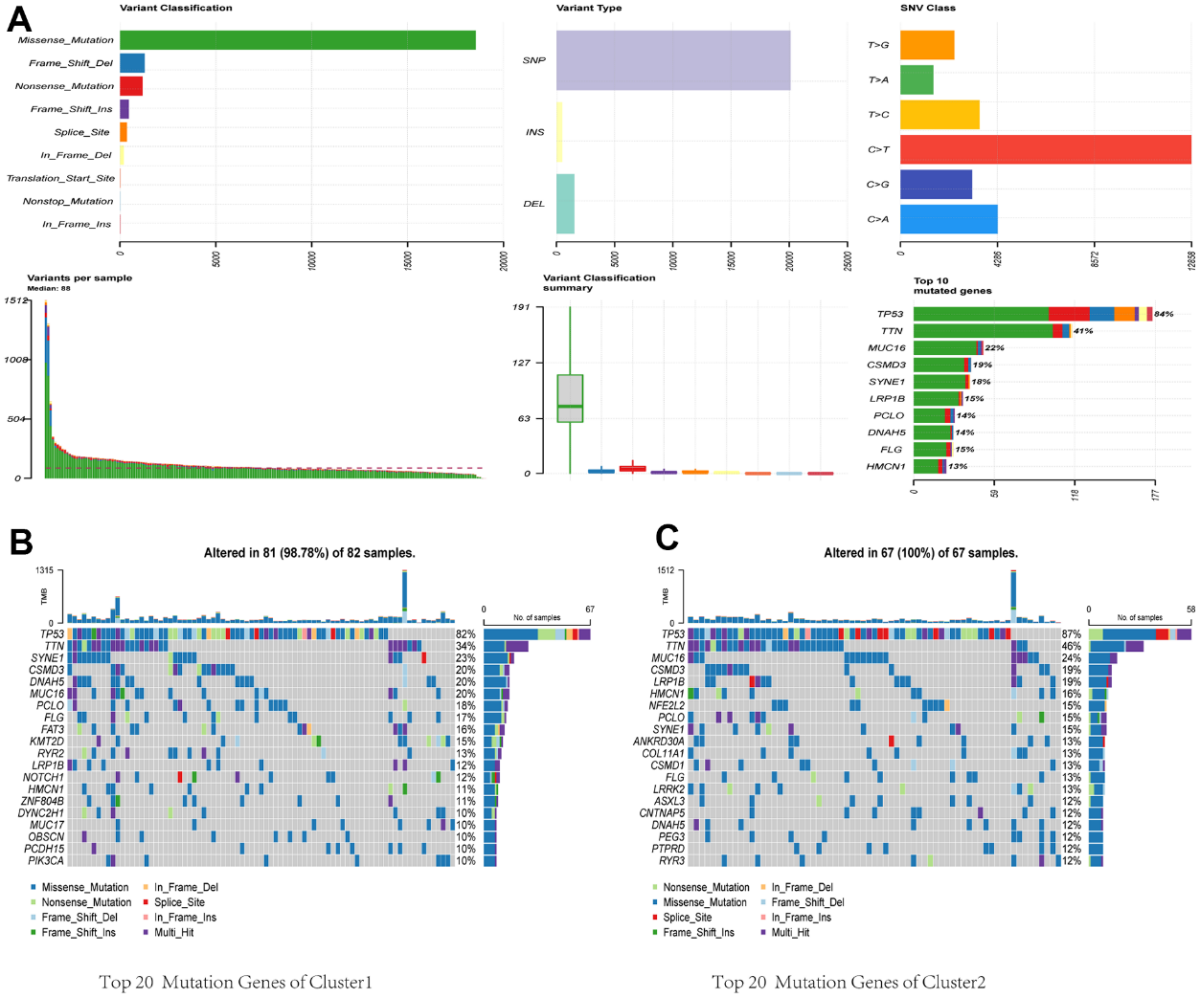
**Comparative analysis of mutational landscapes between two clusters.** (**A**) Overall mutational profile. Mutational landscape of Cluster 1 (**B**) and Cluster 2 (**C**).

We acquired and analyzed mutation data from the TCGA database to study CRGs. Figure 4A shows that missense mutations are the most common variant. Among various types of variants, single nucleotide polymorphisms (SNPs) were the most common, with C > T being the predominant class of single nucleotide variant (SNV). TP53 notably showed the highest mutation frequency among the CRGs. Considering the substantial role of gene mutations in carcinogenesis, we delved into the distribution of somatic mutations in both Cluster 1 and Cluster 2. Figure 4B, 4C depict the corresponding results, showing that Cluster 1 had a somatic mutation rate of 98.78% (81 out of 82 samples), with missense mutations as the primary characteristic. Among these mutations, TP53 had the highest mutation frequency at 82%. In Cluster 2, the mutation rate was 100% (67 out of 67 samples), primarily consisting of missense mutations. TP53 exhibited the highest mutation frequency in this cluster, at 87%. TMB has become a potential marker for identifying cancer patients who may benefit from immunotherapy and predicting their therapeutic response to immune checkpoint inhibitors.

### WGCNA and identification of hub genes related with cuproptosis and immunity

**Figure 5:**
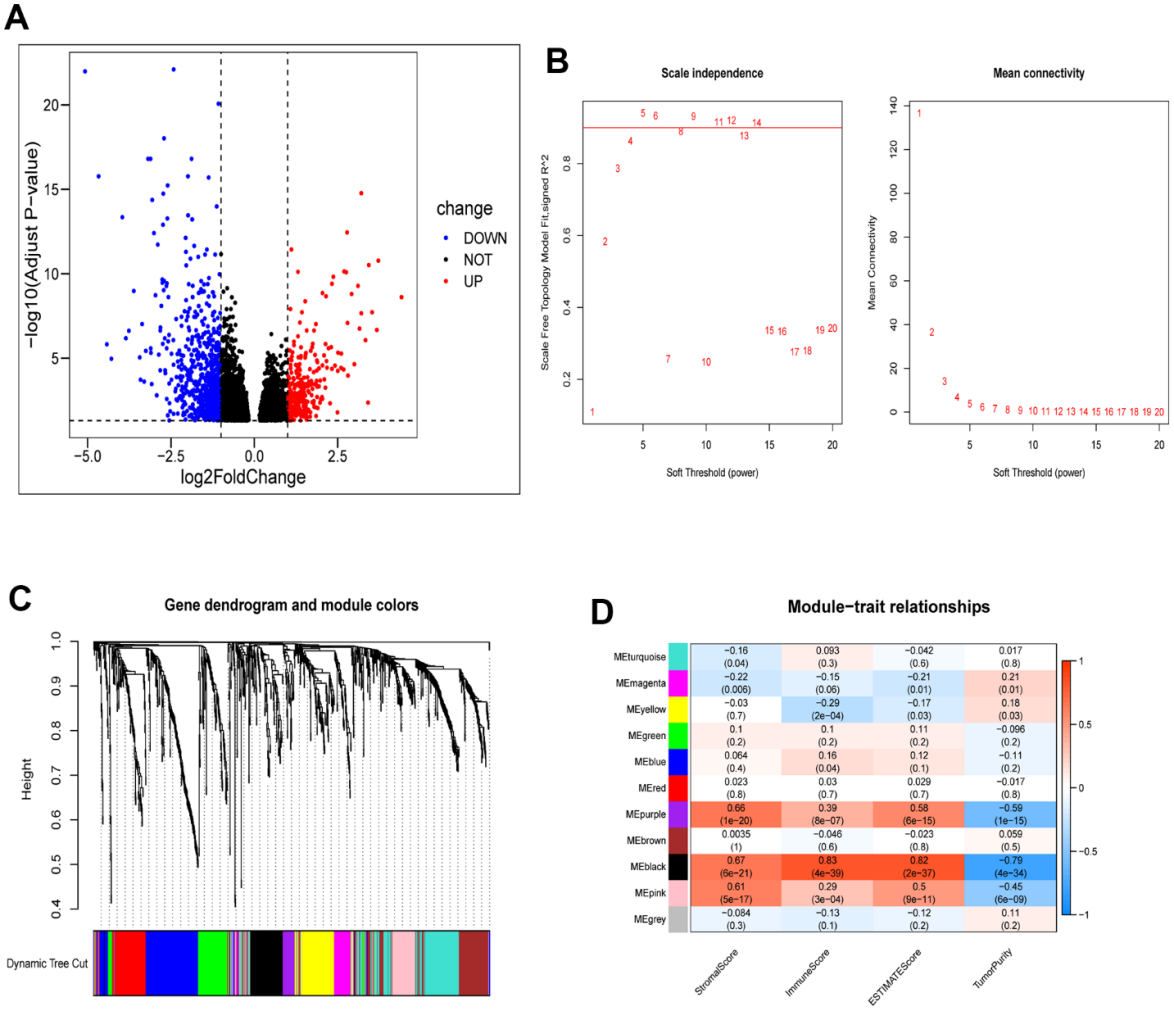
**Identification of module genes associated with clustering and immunity in the WGCNA.** (**A**) Volcano plot depicting differential analysis. (**B**) Analysis of network topology using soft powers. (**C**) Gene dendrogram with module colors. (**D**) Heatmap depicting the relationship between module eigengenes, clusters, and ESTIMATE results.

We identified 987 differentially expressed genes, with 313 upregulated and 674 downregulated, between the two clusters. Their distribution was visualized using a volcano plot (Figure 5A). These genes were then subjected to WGCNA to investigate their co-expression patterns (Figure 5B, 5C). To identify a module associated with both cuproptosis and immunity, we conducted a correlation analysis between the modules and relevant traits (Figure 5D). From the available modules, we selected the black module due to its higher scores in stromal, immune, and estimate fractions, and lower tumor purity.

### Functional enrichment of hub genes and their correlation with immune infiltration

**Figure 6:**
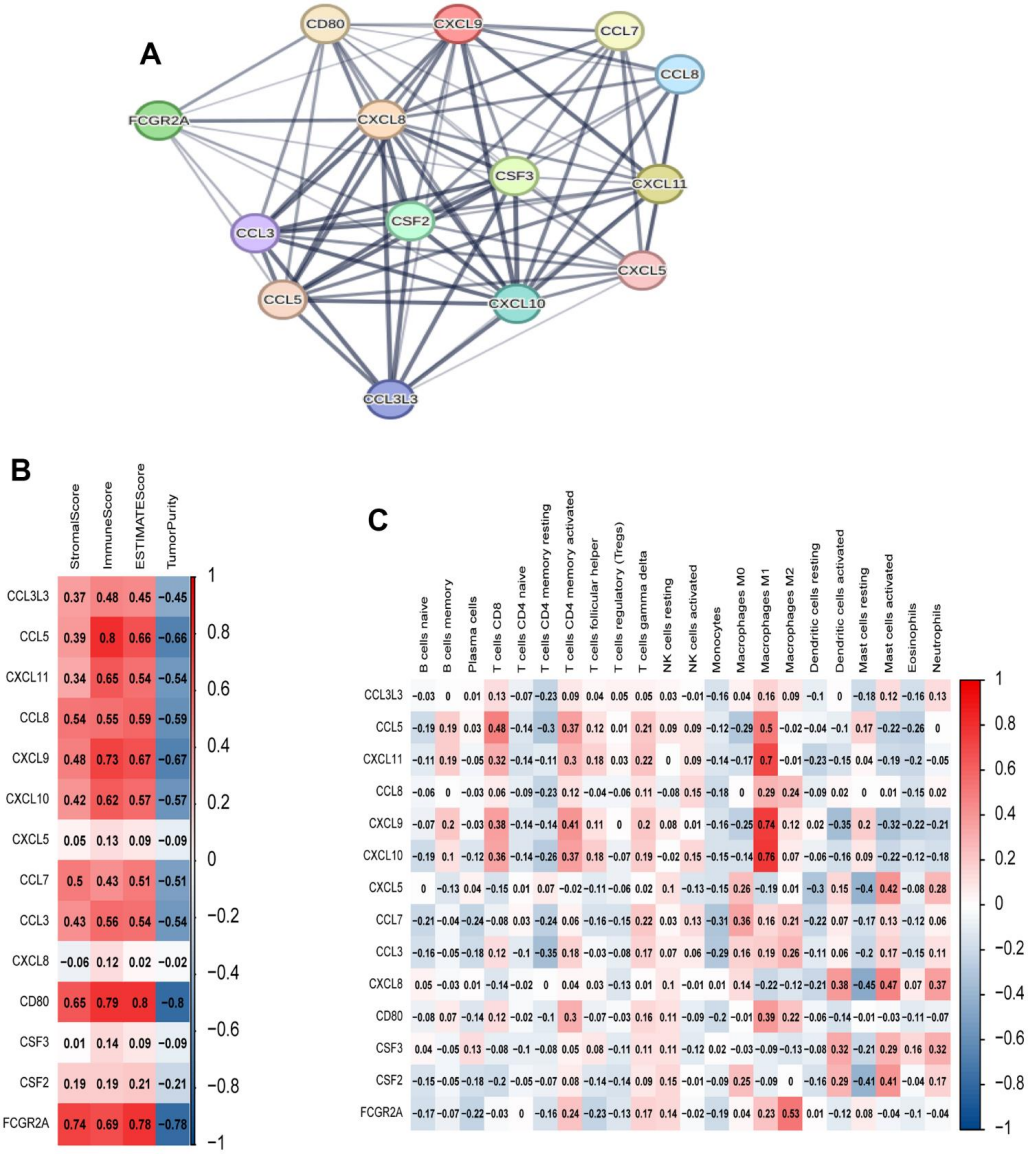
**Analysis of 14 hub genes.** (**A**) PPI network of hub genes. (**B**) Correlation between hub genes and ESTIMATE results. (**C**) Correlation between hub genes and immune cell expression (ssGSEA).

To investigate the relationships among genes within the black module, we constructed a PPI network using the online tool provided on the STRING website. Figure 6A shows the strong associations among the 14 hub genes, which include CCL3L3, CCL5, CXCL11, CCL8, CXCL9, CXCL10, CXCL5, CCL7, CCL3, CXCL8, CD80, CSF3, CSF2, and FCGR2A. Additionally, we performed Spearman’s correlation analysis to investigate the relationships between these genes and immune infiltration, as evaluated using ESTIMATE and ssGSEA. The results indicated significant associations between the majority of genes and the immune response (Figure 6B, 6C).

### GEO validation of immune characteristics between two clusters

**Figure 7:**
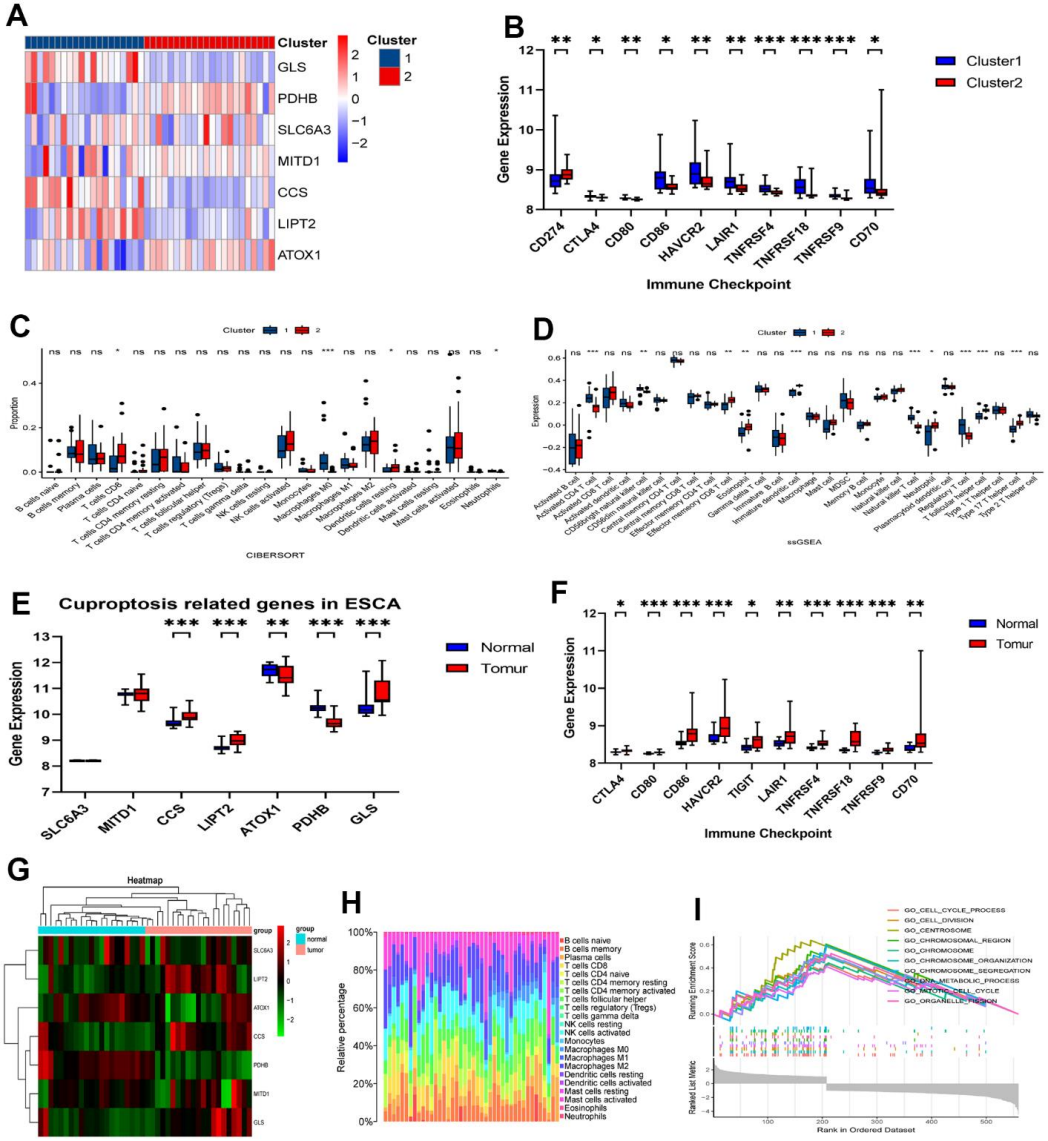
**Validation of immune contexture in GSE199967 between two clusters.** (**A**) Division of GSE199967 patients into two clusters based on 7 CRGs. (**B**) Comparative analysis of targets of immunomodulatory drugs. (**C**) Proportion of immune cells and (**D**) expression of immune cells between the two clusters. (**E**) Comparative analysis of expression of 7 CRGs between tumor and normal tissues in GSE199967. (**F**) Comparative analysis of targets of immunomodulatory drugs between tumor and normal tissues in GSE199967. (**G**) Heatmap in GSE199967. (**H**) Percentages of immune cell types in GSE199967. (**I**) GSEA of the 7 CRGs. ns, not significant, *P < 0.05, **P < 0.01, ***P < 0.001.

Initially, we employed the same clustering approach to partition 42 esophageal cancer samples from the GSE199967 dataset into two clusters, mirroring the TCGA analysis (Figure 7A). We subsequently noted a comparable distribution of CRGs’ expression levels in these two clusters, which resembled the TCGA dataset’s findings. Subsequently, we evaluated the expression levels of immunomodulatory targets and the degree of immune infiltration using the CIBERSORT and ssGSEA methods (Figure 7B–7D). Cluster 1 notably exhibited significantly higher immune system activity in comparison to Cluster 2. Furthermore, we examined the expression of genes related to CRGs and immune checkpoint inhibitors in the GSE199967 cohort. We observed elevated expression levels of these genes in tumors compared to normal tissues (Figure 7E, 7F). We conducted a comprehensive evaluation of the potential implications of CRGs in immunotherapy and immune-related mechanisms. Our analysis showed that macrophages, including M0, M1, and M2 subsets, made up a significant portion of the GEO cohort (Figure 7G, 7H). In order to understand the mechanisms behind the various clinical risks associated with abnormal CRGs expression, we performed a GSEA analysis. The results revealed abnormal enrichment of pathways related to chromosomes and chromosome metabolism (Figure 7I).

### External validation of 7 signature-associated genes

**Figure 8:**
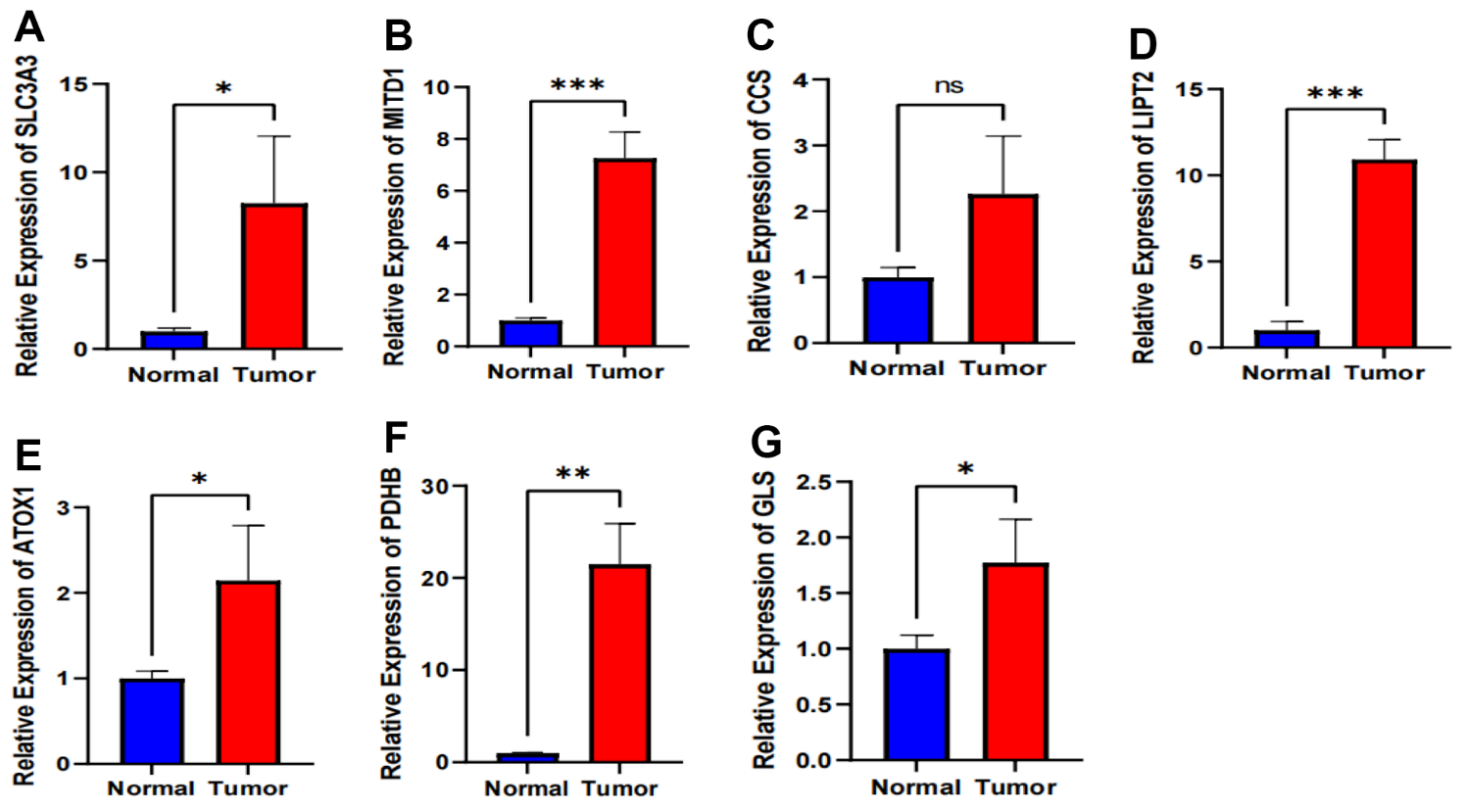
**Validation of expression of 7 CRGs in ESCA tissues using qRT-PCR.** (**A**–**G**) The expression levels of the 7 CRGs in 10 pairs ESCA tissues and corresponding adjacent normal tissues were examined by qRT-PCR. ns, not significant, *P < 0.05, **P < 0.01, *** *P* < 0.001.

We performed qRT-PCR experiments to assess the expression levels of the 7 signature-associated genes in both esophageal cancer tissue and normal tissue. The results show a significant increase in the expression of SLC3A3, MITD1, LIPT2, ATOX1, PDHB, and GLS in tumor tissues compared to normal esophageal tissues (Figure 8A, 8B, 8D–8G). Of the six genes analyzed, PDHB showed an expression trend opposite to the decreasing trend observed in the TCGA database, while the other five genes exhibited a similar increasing trend as seen in the TCGA database. The expression level of CCS showed no significant difference between esophageal cancer and adjacent tissues (Figure 8C). The gene expression results from the clinical tissue samples closely matched the RNA sequencing data analyzed in the TCGA database.

### Prognosis prediction based on CRGs

**Figure 9:**
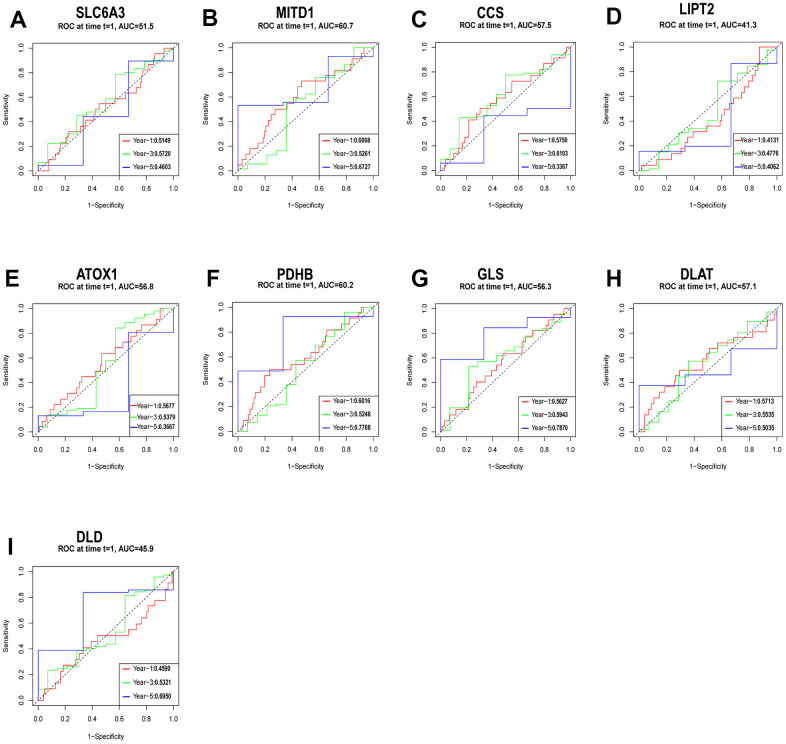
Evaluation of the independent prognostic value of gene expression using timeROC curves for 1-, 3-, and 5-year overall survival (OS) predictions of SLC6A3 (**A**), MITD1 (**B**), CCS (**C**), LIPT2 (**D**), ATOX1 (**E**), PDHB (**F**), GLS (**G**), DLAT (**H**), and DLD (**I**) through the nomogram in the TCGA cohort.

We used a cohort of 151 patients with the TCGA-ESCA for external validation. Before conducting further analysis, we standardized the gene expression data using the “sva” package. Using relevant hub genes as predictive variables and taking the median risk score as a reference, we assessed the survival index of patients based on their outcome time and whether the outcome indicated death or survival (Figure 9A–9I). The ROC analysis showed the strong predictive ability of specific hub genes. Particularly, MITD1 displayed a positive predictive impact on the risk score, as supported by its AUC values of 0.6068 at 1 year, 0.5261 at 3 years, and 0.6727 at 5 years (Figure 9B). Similarly, PDHB and GLS exhibited strong predictive capabilities, with PDHB achieving AUC values of 0.6016 at 1 year, 0.5248 at 3 years, and 0.7788 at 5 years, and GLS achieving AUC values of 0.5627 at 1 year, 0.5943 at 3 years, and 0.7870 at 5 years (Figure 9F, 9G).

### Differential expression of CRGs in different pathologic stages and histological grades of ESCA

**Figure 10:**
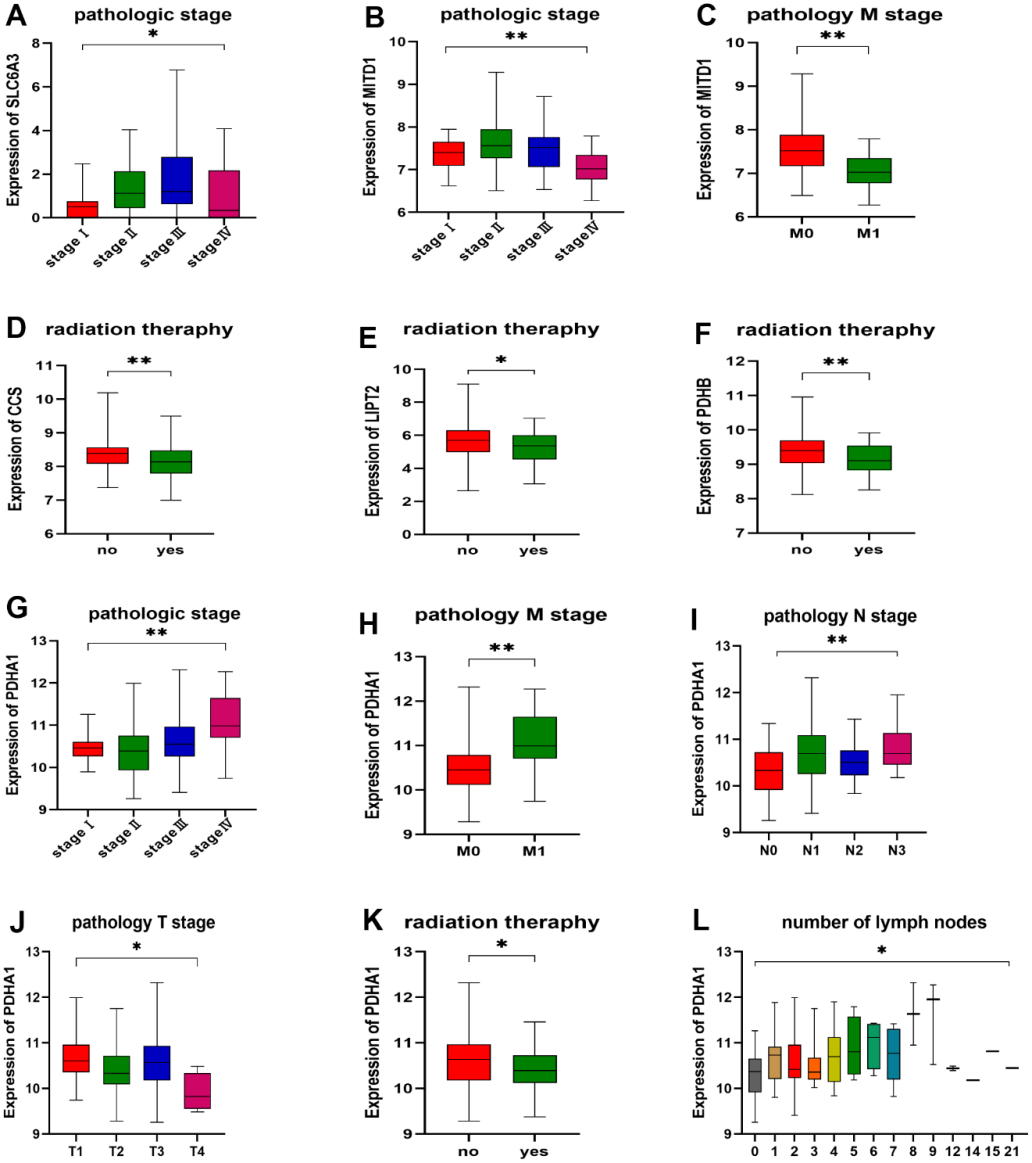
**Correlation between gene expression and clinicopathological staging characteristics.** (**A**) Expression of SLC6A3 in different pathologic stages. (**B**, **C**) Expression of MITD1 in different pathologic stages and pathology M stage. (**D**) Expression of CCS, (**E**) LIPT2, and (**F**) PDHB in different radiation therapy. (**G**) Expression of PDHA1 in different pathologic stages, (**H**) pathology M stage, (**I**) pathology N stage, (**J**) pathology T stage, (**K**) radiation therapy, and (**L**) number of lymph nodes. *P < 0.05, **P < 0.01.

As illustrated in Figure 10, the expression levels of three genes, namely SLC6A3, MITD1, and PDHA1, exhibited variations across different pathological stages (Figure 10A, 10B, 10G). Similarly, four genes, namely CCS, LIPT2, PDHB, and PDHA1, displayed variations in response to different radiation therapies (Figure 10D–10F, 10K). Of these genes, MITD1 and PDHA1 exhibited variations in different pathological M stages of ESCA. Specifically, the expression levels of SLC6A3, MITD1, and PDHA1 exhibited an increasing trend with tumor pathological stage, whereas CCS, LIPT2, PDHB, and PDHA1 displayed a decreasing trend in response to radiation therapy. However, MITD1 and PDHA1 displayed a contrasting trend in relation to the pathological M stage. Furthermore, the expression levels of PDHA1 varied across distinct pathological N stages, pathological T stages, and the count of metastatic lymph nodes in ESCA. Importantly, an increasing trend was observed in different pathological N stages and the count of metastatic lymph nodes, while a decreasing trend was observed in pathological T stages. These findings indicate a potential link between the expression of CRGs, disease grade, and radiotherapy resistance in ESCA. Patients with esophageal cancer were categorized into two groups, namely high expression and low expression, based on their PDHA1 expression. The Kaplan-Meier curve consistently showed that the high expression group had significantly shorter overall survival than the low expression group (Supplementary Figure 3).

## DISCUSSION

This study is a pioneering investigation into the link between CRGs and ESCA. The main objective was to comprehensively analyze the expression patterns and immune infiltration of seven specific CRGs in patients with ESCA. Our analysis successfully identified two distinct molecular clusters based on the expression profiles of these CRGs. To clarify the functional differences between these clusters, we performed GSEA using all differentially expressed genes between Cluster 1 and Cluster 2. Our GSEA results unveiled several significant immunity-related pathways, including the humoral immune response. Importantly, we created a prognostic score that includes seven crucial CRGs (SLC6A3, MITD1, CCS, LIPT2, ATOX1, GLS, and PDHB). Of these genes, SLC6A3 has gained recognition as a risk factor due to its association with various familial mutations related to neuropsychiatric and neurological disorders [22]. Moreover, MITD1 deficiency has been discovered to hinder the growth and migration of clear cell renal cell carcinoma by inducing ferroptosis through the TAZ/SLC7A11 pathway [23]. Michael Grasso’s previous research showed that the copper chaperone for superoxide dismutase, CCS, binds temporarily to MEK1, promoting copper loading and thereby increasing MEK1/2 kinase activity [24]. The mechanism by which LIPT2 presence outside the mitochondrion induces apoptotic cell death is likely associated with mitochondrial dysfunction [25]. Inside human cells, ATOX1 plays a vital role in copper transport to the secretory pathway, where it transfers copper to copper-transporting ATPases (ATP7A and ATP7B) found in the trans-Golgi network and different endocytic vesicles. This mechanism promotes the maturation of copper-dependent enzymes in the secretory pathway and sustains copper levels in the cytosol and mitochondria [26]. Succinylation of GLS at K311 increases GLS activity, promoting glutaminolysis and generating NADPH and glutathione to counteract oxidative stress-induced reactive oxygen species (ROS) and apoptosis. As a result, this process facilitates tumor growth [27]. Manipulating PDHB expression through knockdown or overexpression resulted in decreased or increased expression of Myf5, MyoD, MyoG, and MyHC, resulting in reduced or increased rates of myogenic differentiation. Additionally, overexpressing PDHB in skeletal muscle alleviated D-galactose-induced sarcopenia in mice [28].

We assessed the immune characteristics of the two identified clusters using various methodologies, including ESTIMATE, CIBERSORT, and ssGSEA. ESTIMATE analysis showed that Cluster 1 had higher stromal, immune, and overall ESTIMATE scores, indicating a more dynamic tumor immune microenvironment. CIBERSORT analysis showed a significant increase in the proportion of CD4 memory activated T cells and resting NK cells in Cluster 1. Prior studies have emphasized that naive CD4 T lymphocytes differentiate into effector/memory cells during conventional adaptive immune responses upon recognizing foreign antigens [29]. In the immunosuppressive tumor microenvironment, NK cells may experience dysfunction due to exposure to inhibitory molecules produced by cancer cells, contributing to tumor escape [30]. Furthermore, the ssGSEA analysis indicated higher expression of 25 immune cell subtypes in Cluster 1, including CD8 T cells, CD4 T helper cells, dendritic cells (DCs), natural killer T (NKT) cells, regulatory T cells, and MDSCs. The infiltration of these immune cells has a significant impact on the clinical characteristics of ESCA. Higher intratumoral infiltration of CD8 T cells has been linked to longer survival times [31]. Th1 cells have been demonstrated to suppress ESCC cell proliferation, increase chemosensitivity and radiosensitivity, and correlate with improved prognosis [32]. DCs play a critical role in enhancing anti-tumor immunity by activating T cells [33]. Conventional type 1 dendritic cells are recruited into the tumor microenvironment upon being stimulated by NK cells [34]. Infiltration of LAMP-3-expressing DCs is positively correlated with intratumoral CD8 T cell levels and is linked to a favorable prognosis in ESCC [35]. NKT cells contribute to anti-tumor immunity by rejuvenating exhausted CD8 T cells in a tumor model resistant to anti-PD-1 therapy [36]. Macrophages are commonly categorized into M1 (proinflammatory; anti-tumor) and M2 (anti-inflammatory; tumor-promoting) subtypes [37]. Cluster 1 showed a higher proportion of M1 subtype macrophages, indicating a potential promotion of anti-tumor Th1-type responses, whereas Cluster 2 tended to establish a tolerogenic microenvironment. In summary, our analysis of the immune contexture showed increased immune cell infiltration in Cluster 1, suggesting higher immunological competence and the potential for immunotherapy benefits.

Previous studies extensively reported the FDA approval of ICIs that target programmed cell death 1 (PD-1), programmed cell death 1 ligand, and cytotoxic T lymphocyte antigen 4 [38]. Additionally, co-inhibitory receptor targets, like lymphocyte activation gene-3, T cell immunoglobulin-3, and T cell immunoglobulin and ITIM domain, have been identified as well [39]. This study aimed to compare two clusters of immunomodulatory drugs that have been studied in clinical trials for metastatic ESCC [40]. Importantly, Cluster 1 showed significantly higher expression levels of most of these targets. Subsequently, we analyzed the mutational profiles within the two clusters, revealing a significant difference between them. Specifically, Cluster 2 had a higher TMB compared to Cluster 1. TMB plays a critical role in influencing the generation of immunogenic peptides and, consequently, impacting the response to immunotherapy [41]. Therefore, these findings suggest that Cluster 1 may show a more favorable response to immunotherapy.

Subsequently, we conducted WGCNA to identify the blue module that exhibited distinct characteristics related to both CRGs and immune scores. By assessing module membership and gene significance values, we identified 14 genes within this module, which included CD80. CD80, among these genes, is associated with “professional antigen-presenting cells” that initiate T-cell activation via antigen presentation. Conversely, insufficient CD80 costimulation during antigen presentation may result in tolerance induction, and inhibiting CD80 costimulation has shown the potential to hinder the progression of autoimmune diseases in multiple animal models [42]. These occurrences are likely to be widespread in Cluster 1. Consequently, we hypothesize that Cluster 1 may demonstrate a more favorable response to immunotherapy than Cluster 2.

Cluster 1 exhibited “hot” tumor characteristics in its immunological features, while Cluster 2 showed characteristics indicative of a “cold” tumor [43]. By verifying CRGs expression in 10 pairs of cancer and normal tissues through qRT-PCR, we observed that Cluster 2 exhibited the overall immunological characteristics of ESCA, indicating a reduced immune response. It is plausible that CRGs may contribute to the conversion of a cold tumor to a hot tumor in ESCA. Furthermore, we developed a prognostic model based on CRGs and examined their expression in various pathological stages and histological grades of ESCA. Several hub genes showed robust predictive capabilities, as revealed by ROC analysis. Notably, PDHA1 expression displayed variations across distinct pathological N stages, T stages, and counts of metastatic lymph nodes in ESCA. Increasing trends were observed in N stages and lymph node count, whereas decreasing trends were noticed in T stages.

This study has contributed to our comprehension of the connection between CRGs and immunity; nonetheless, it’s important to recognize specific constraints. Firstly, the retrospective nature of the study underscores the importance of prioritizing future research on prospective studies, which can mitigate potential biases linked to retrospective designs. Furthermore, the lack of datasets with a sample size large enough and encompassing clinical prognostic information hampers the further validation of the results. Finally, the prognostic signature was built and confirmed using data from public databases. However, depending solely on TCGA and GEO databases restricts our capacity to explore CRGs’ expression at the protein level and reveal the direct mechanisms by which they influence anti-tumor immunity. Thus, it’s essential to collect more biological evidence to supplement the statistical results and enhance the robustness of the findings. Future research efforts should concentrate on unraveling the direct mechanisms that underlie the observed phenomena.

## MATERIALS AND METHODS

### Data sources and preprocessing

Transcriptome profiling data, including HTSeq-Counts and HTSeq-FPKM, and clinical information, were acquired from the TCGA-ESCA project using R and the R package “TCGAbiolinks” [44]. The download was executed to collect extensive clinical data, which included age, sex, T stage, N stage, M stage, and prognostic information. Only cases with complete clinical information were included in the subsequent analyses. To further investigate, a log2(FPKM+1) transformation was applied to the Level 3 HTSeq-FPKM data of 173 primary solid tumor samples, while HTSeq-Counts were used for differential analysis.

To obtain nucleotide variation data, specifically MuTect2, for the 173 patients with ESCA, we used the R package “maftool” [45]. Mutational landscape analysis was exclusively conducted on these 173 patients due to the absence of mutation information for some ESCA patients. Waterfall plots, created with the R package “ComplexHeatmap” [46], were used to depict the genetic mutations observed in these patients. The tumor mutation burden (TMB) was calculated as the number of mutations per megabase using data on single nucleotide variations.

Expression profiling data from the GSE199967 array were obtained from the Gene Expression Omnibus (GEO) database. Subsequently, we extracted 27 cuproptosis-related genes (CRGs) from the reports by Tsvetkov [14] and the MsigDB. A total of 27 CRGs were included in the analysis conducted for this study (Supplementary Table 1). Seven specific CRGs were filtered based on the comparison of expression levels between normal tissues and ESCA samples.

### Immune infiltration analysis

The ESTIMATE method, which analyzes gene expression patterns in tumor samples, quantified stromal and immune cell proportions in ESCA patients to evaluate the tumor microenvironment (TME). This assessment included the evaluation of stromal score (reflecting stromal content), immune score (indicating the extent of immune cell infiltration), ESTIMATE score (a composite measure of stroma and immune components), and tumor purity. These analyses were conducted using the R package “estimate” [47].

To estimate cell composition, we used the CIBERSORT computational method, which analyzes gene expression profiles. The deconvolution algorithm was applied to determine the relative abundance of 22 immune cell types in each ESCA patient [48]. The sum of the fractions of these 22 immune cell types in each sample equaled 1.

To assess immune cell type infiltration levels, we used the ssGSEA method from the R package “GSVA” [49]. This method allowed us to evaluate the expression profiles of 28 published gene sets specific to immune cells, giving insights into the infiltration levels of these 28 immune cell types [50].

### Consensus clustering analysis of CRGs and GSEA analysis

We extracted the expression profiles of CRGs and conducted coherent clustering with the “ConsensusClusterPlus”| R package [51]. This analysis resulted in the samples being partitioned into two distinct clusters. Additionally, we identified consensus molecular subtypes (CMS) for each sample using the “CMScaller” R package [52]. CMS is a robust classification system for ESCA with distinct features: CMS1 (immune), CMS2 (canonical), CMS3 (metabolic), and CMS4 (mesenchymal) [53]. We employed a Sankey diagram to visualize the relationship between the two clusters and CMS.

Next, we performed gene set enrichment analysis (GSEA) on the differentially classified risk groups using the “ggplot2” and “clusterProfiler” packages in R software [54]. Pathway enrichment was deemed significant when it met specific criteria, including a normalized enrichment score (|NES| > 1), a P-value < 0.05, and a false discovery rate (FDR) q-value < 0.05.

### Differential expressed genes

Expression profiling data (HTSeq-Counts) were analyzed with the “DESeq2” R package [55] to identify differentially expressed genes (DEGs) between the two clusters. The criteria for selecting DEGs included a threshold of |log2FoldChange| > 1 and an adjusted P-value < 0.05.

### Weighted gene co-expression network analysis and functional enrichment analysis

Weighted gene co-expression network analysis (WGCNA) was performed on the differentially expressed genes with the “WGCNA” R package [56]. To ensure the construction of a co-expression network with a scale-free distribution, we chose a soft power of 5. Eleven modules were identified, and we assessed their associations with the cluster, stromal score, immune score, ESTIMATE score, and tumor purity. Subsequently, we identified 14 genes based on calculations of module membership (MM) and gene significance (GS).

We performed Gene Ontology (GO) analysis using the “clusterProfiler” R package [54] to gain insights into the functions of the 14 selected DEGs. Additionally, we constructed a protein-protein interaction (PPI) network using the STRING database [57]. Furthermore, we used the “corrplot” R package to conduct Spearman’s correlation analysis, investigating the relationships between genes, gene-ESTIMATE, and gene-ssGSEA.

### Tissue samples and qRT-PCR

We obtained 10 tumor tissue samples and corresponding adjacent normal esophageal tissue samples from patients who underwent tumor resection for ESCA. The tissue sample collection took place at the Thoracic Surgery Department of the Second Hospital of Hebei Medical University, with approval from the hospital’s Medical Ethics Committee. To maintain sample integrity, fresh tumor and non-tumor tissues were promptly frozen in liquid nitrogen. RNA extraction utilized the TRIzol Reagent (Invitrogen, USA). cDNA synthesis was conducted using the PrimeScriptTM RT reagent Kit with gDNA Eraser (Takara, Beijing, China) via reverse transcription. Quantitative real-time polymerase chain reaction (qRT-PCR) analysis utilized the SYBR Premix Ex Taq (Takara). Expression data were normalized to the internal control GAPDH using the 2^−ΔΔCT^ method. Gene-specific primers were synthesized by Sangon Biotech (Shanghai, China), and their sequences are available in Supplementary Table 2.

### Prognosis prediction of CRGs and difference analysis of scores with clinical stages

Using the median risk score as the basis, we employed key hub genes as predictive variables to estimate patients’ survival index, taking into account their survival time and outcome (death or survival). Furthermore, we used the signature to calculate the 1-year, 3-year, and 5-year survival rates through the nearest neighbor estimation method [58]. To evaluate its predictive performance, we generated receiver operating characteristic (ROC) curves with the “survivalROC” R package. The clinicopathological data of ESCA samples were retrieved from the TCGA database. Statistical analysis was conducted using the R programming language. The significance was assessed by employing either the Wilcoxon rank sum test or the Kruskal-Wallis rank sum test for comparisons, depending on the number of clinical stages.

### Statistical analysis

All statistical analyses were performed using R software version 4.2.1. Figures were created and compiled using Adobe Illustrator. The Wilcoxon rank-sum test was used for box plot analyses. Spearman’s coefficient was used for correlation analysis. The chi-square test was used to compare clinical characteristics between the two clusters, and Fisher’s exact test was used when necessary. Multivariate logistic regression analysis was performed to assess the impact of clinical characteristics on the clusters. All hypothesis tests were two-sided, and statistical significance was defined as a P-value less than 0.05.

## Supplementary Material

Supplementary Figures

Supplementary Tables
