# Supplementary material for: Potential impact of cuproptosis-related genes on tumor immunity in esophageal carcinoma
Source: Aging (Albany NY). 2023 Dec 30;15(24):15535–56. doi: 10.18632/aging.205391 (PMC10781504; doi:10.18632/aging.205391)
Supplement: Supplementary Figures [file aging-15-205391-s001.pdf]

SUPPLEMENTARY FIGURES

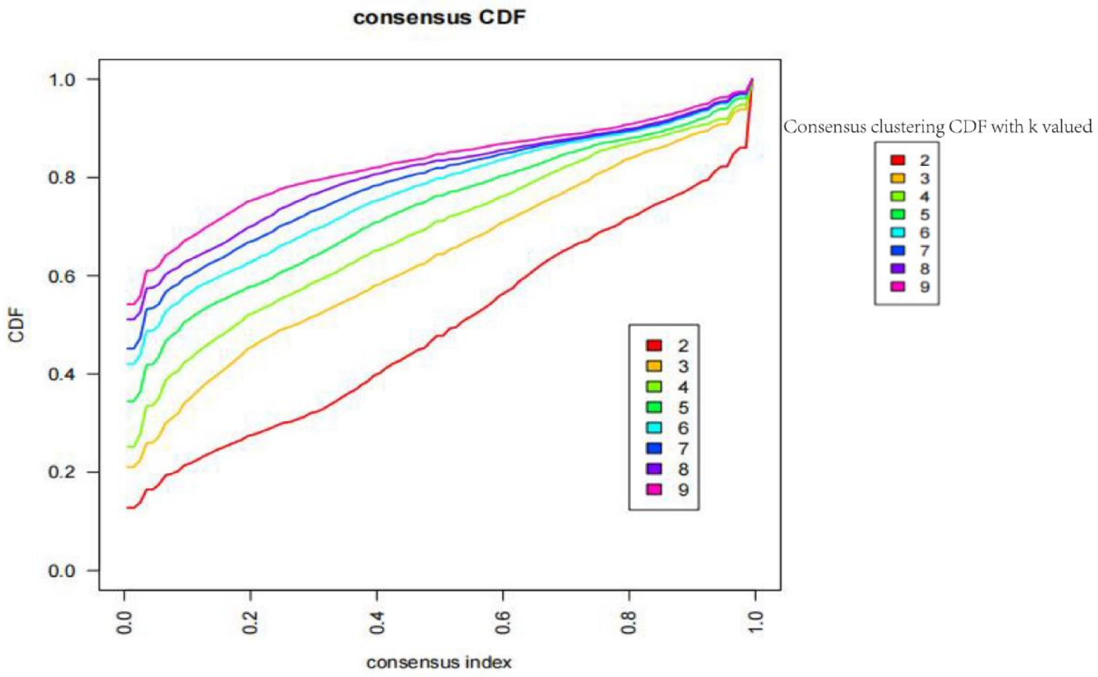

Consensus clustering CDF with k valued 2 to 9

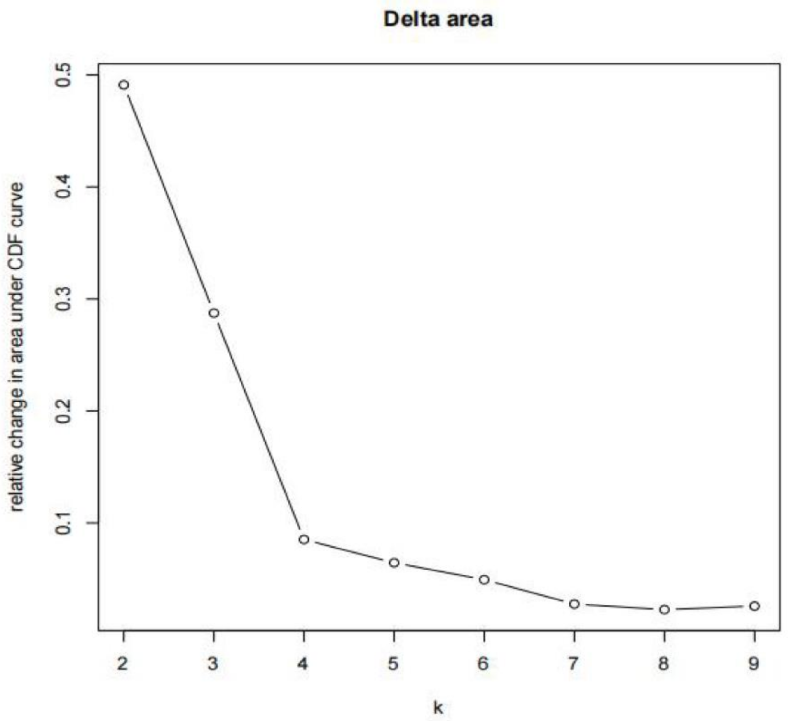

Relative change in area under the CDF curve for k = 2 through 9

Supplementary Figure 1. Heatmap corresponding to the consensus matrix using consensus clustering.

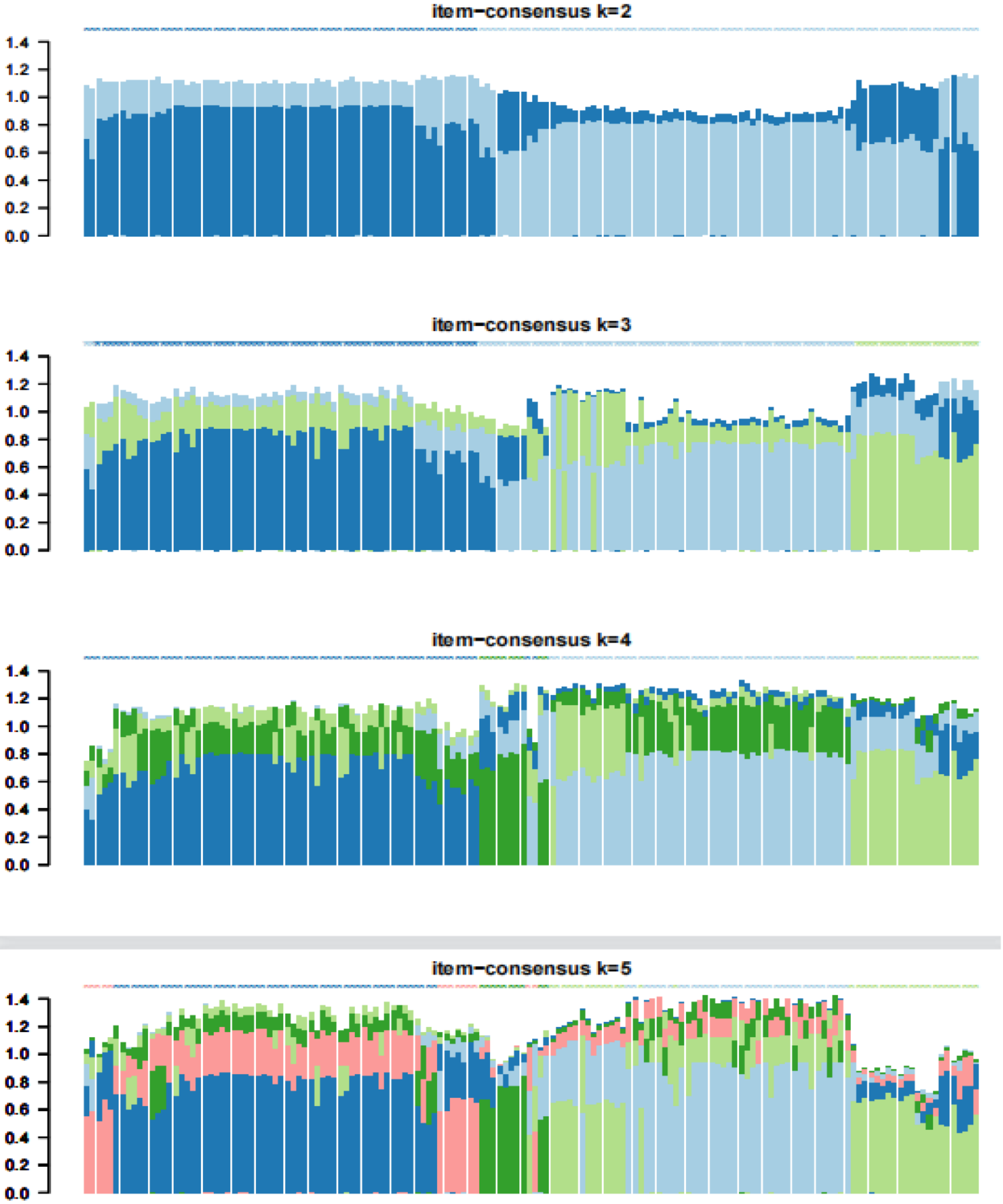

Supplementary Figure 2. Heatmap corresponding to the item-consensus using consensus clustering.

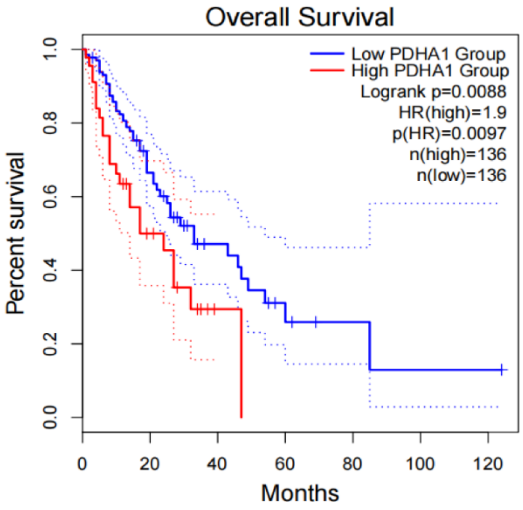

Supplementary Figure 3. Survival analysis of PDHA1.
