# Supplementary material for: Potential impact of cuproptosis-related genes on tumor immunity in esophageal carcinoma
Source: Aging (Albany NY). 2023 Dec 30;15(24):15535–56. doi: 10.18632/aging.205391 (PMC10781504; doi:10.18632/aging.205391)
Supplement: Supplementary Tables [file aging-15-205391-s002.pdf]

SUPPLEMENTARY TABLES

Supplementary Table 1. 27 cuproptosis-related genes (CRGs) were extracted from the reports by Tsvetkov and the MsigDB.

27 cuproptosis-related genes (CRGs)		
SLC25A5	CP	SLC23A2
NDUFB2	DLD	PDHX
DLST	LIAS	ATP7B
NDUFA1	COX7B	NDUFA2
SLC31A1	FDX1	SLC6A3
LIPT1	DLAT	PIH1D2
MITD1	ATP7A	CCS
LIPT2	ATOX1	NDUFB1
SLC22A5	PDHB	GLS

Supplementary Table 2. 7 CRGs specific primers.

7 CRGs specific primers		
**CRGs**	**Forward primer (5’-3’)**	**Reverse primer (5’-3’)**
SLC6A3	GAGAGAACACGAACAAACC	TTACAAACACAAGACA
MITD1	UAGCGCUGGACCGGTCA	AGCTCACGGAGTGGTTCAACT
CCS	GGGAACTATTGACGGCCTGG	GTCAGCATCAGCACGGACAT
LIPT2	CGTGGTTTGAGCACATCG	AAGGCCACAAGGAAAGGTG
ATOX1	GTGCTGAAGCTGTCTCTCGG	GCCCAAGGTAGGTAGGAAACAGTCTTT
PDHB	CTCAGCACTCGCAATGCTTC	AAGTCCTTTCGCATCCTCGG
GLS	AGGGTCTGTTACCTAGCTTGG	ACGTTCGCAATCCTGTAGATTT
GAPDH	AGGTCGGTGTGAACGGATTTG	TGTAGACCATGTAGTTGAGGTCA
